# Evidence for Cardiorenal Protection with SGLT-2 Inhibitors and GLP-1 Receptor Agonists in Patients with Diabetic Kidney Disease

**DOI:** 10.3390/jpm12020223

**Published:** 2022-02-06

**Authors:** Panagiotis I. Georgianos, Vasilios Vaios, Stefanos Roumeliotis, Konstantinos Leivaditis, Theodoros Eleftheriadis, Vassilios Liakopoulos

**Affiliations:** 1 Section of Nephrology and Hypertension, 1st Department of Medicine, AHEPA Hospital, Aristotle University of Thessaloniki, GR54636 Thessaloniki, Greece; pangeorgi@yahoo.gr (P.I.G.); vvaios_85@yahoo.gr (V.V.); st_roumeliotis@hotmail.com (S.R.); konleiv@windowslive.com (K.L.); 2 Department of Nephrology, School of Medicine, University of Thessaly, GR4110 Larissa, Greece; teleftheriadis@yahoo.com

**Keywords:** SGLT-2 inhibitors, GLP1-RA, diabetic kidney disease, cardiorenal protection, randomized controlled trials

## Abstract

For almost two decades, the management of patients with type 2 diabetes mellitus (T2DM) and chronic kidney disease (CKD) was based on the optimal glycemic and blood pressure control as well as on the adequate blockade of the renin-angiotensin-system. Over the past few years, sodium-glucose co-transporter 2 (SGLT-2) inhibitors and glucagone-like peptide 1 receptor agonists (GLP1-RAs) were added to our therapeutic armarhatum, offering promise for more effective mitigation of the substantial residual cardiorenal risk of these patients. Large randomized controlled trials (RCTs) designed to demonstrate the cardiovascular safety of SGLT-2 inhibitors and GLP1-RAs showed that these novel anti-diabetic medications improve cardiovascular outcomes in patients with T2DM. RCTs conducted specifically in CKD patients with or without T2DM demonstrated that SGLT-2 inhibitors were also effective in retarding the progression of kidney injury to end-stage kidney disease. The kidney protective effects of GLP1-RA are not yet proven, but RCTs are currently ongoing to investigate this crucial research question. In this article, we review the available clinical-trial evidence supporting the use of SGLT-2 inhibitors and GLP1-RAs for cardiorenal protection in patients with T2DM and CKD. We provide clinical practice recommendations for a personalized approach in the use of these novel therapies, according to the severity of CKD and the presence of other cardiometabolic risk factors.

## 1. Introduction

Type 2 diabetes mellitus (T2DM) and chronic kidney disease (CKD) are two conditions with a growing prevalence globally [1]. T2DM and CKD often co-exist; it is estimated that approximately 40–50% of patients with T2DM have some degree of renal impairment, defined as persistent albuminuria ≥ 30 mg/g, persistently reduced estimated-glomerular-filtration-rate (eGFR) < 60 mL/min/1.73 m^2^ or both [2,3]. Injury to the diabetic kidney involves the complex interaction among metabolic, hemodynamic, pro-inflammatory, and pro-fibrotic factors [3]. T2DM is by far the most frequent cause of CKD, accounting for nearly half of all incident cases of end-stage kidney disease (ESKD) requiring hemodialysis or peritoneal dialysis [1]. Even more important is the fact that the presence and severity of CKD significantly shortens the life expectancy of patients with T2DM. In fact, patients with T2DM and CKD are much more likely to die from cardiovascular causes rather than progress to kidney failure [4,5].

From the early 1990s and thereafter, optimal glycemic and blood pressure (BP) control have been two fundamental components of standard-of-care treatment for patients with T2DM and CKD [6,7]. In 2001, two landmark trials, the IDNT (Irbesartan Diabetic Nephropathy Trial) and RENAAL (Reduction of Endpoints in NIDDM with the Angiotensin II Antagonist Losartan), demonstrated that compared with placebo (in IDNT) or active-treatment with a calcium-channel-blocker (in RENAAL), an angiotensin-receptor-blocker was more effective in slowing the progression of CKD in patients with T2DM and overt nephropathy [8,9]. These kidney protective effects were paralleled with a 20–30% reduction in the risk of hospitalization for heart failure (HF) [8,9]. However, despite the primary or secondary prevention of various risk factors and adequate blockade of the renin-angiotensin-system (RAS), the 10-year cumulative rate of all-cause mortality in patients with T2DM and CKD was 31.1%, indicating that the residual cardiorenal risk remained disproportionably high [10]. A large unmet need to identify therapies effective at improving “hard” clinical outcomes and life expectancy in these patients persisted [11].

In this article, we discuss recent advances in the management of diabetic kidney disease with the development and introduction of sodium-glucose co-transporter type 2 (SGLT-2) inhibitors and glucagon-like peptide 1 (GLP-1) receptor agonists, two novel classes of anti-diabetic medications that exert favorable effects on both the heart and the kidney. We review the currently available clinical-trial evidence and provide clinical practice recommendations for a personalized approach in the utilization of these novel therapies in patients with T2DM, taking into consideration the co-existence of CKD and other cardiometabolic risk factors.

## 2. SGLT-2 Inhibitors

SGLT-2 inhibitors lower blood glucose levels by promoting the urinary excretion of sodium and glucose in the proximal tubule [12,13]. The US Food and Drug Administration (FDA) has approved 4 SGLT-2 inhibitors for the improvement of glycemic control in patients with T2DM and preserved kidney function: empagliflozin, canagliflozin, dapagliflozin, and ertugliflozin [12,13]. However, large cardiovascular outcome trials that were designed to demonstrate their safety provided impressive evidence for cardiorenal protection with SGLT-2 inhibition in T2DM patients with or without established atherosclerotic cardiovascular disease (Table 1). In detail, these trials consistently demonstrated the non-inferiority of SGLT-2 inhibitors relative to placebo [14,15,16,17], but the EMPA-REG OUTCOME (Empagliflozin Cardiovascular Outcome Event Trial in Type 2 Diabetes Mellitus Patients) and CANVAS (Canagliflozin Cardiovascular Assessment Study) trials unexpectedly showed that empagliflozin and canagliflozin were superior to placebo in lowering the risk for major adverse cardiovascular events [15,17]. An even more impressive and consistent benefit was the reduction in the risk for HF hospitalization. Secondary analyses of these trials also provided preliminary evidence that SGLT-2 inhibitors may mitigate the risk for worsening of kidney function [14,15,16,17,18].

Since the aforementioned cardiovascular outcome trials included predominantly patients with normal kidney function or mild CKD, the CREDENCE (Canagliflozin and Renal Events in Diabetes with Established Nephropathy Clinical Evaluation) trial was designed to specifically explore the safety and efficacy of canagliflozin (10 mg/day) relative to placebo in 4401 patients with T2DM and overt nephropathy [19]. According to the inclusion criteria, all patients enrolled in CREDENCE had an eGFR of 30 to <90 mL/min/1.73 m^2^ and macroalbuminuria (UACR > 300 mg/g). All patients were also receiving background therapy with a RAS-blocker. This trial was prematurely terminated because canagliflozin provoked an impressive improvement in clinical outcomes. Over a median follow-up of 2.6 years, canagliflozin provoked a placebo-subtracted reduction of 30% in the primary composite outcome of doubling of serum creatinine, ESKD or death from renal and cardiovascular causes [hazard ratio (HR): 0.70; 95% confidence interval (CI): 0.59–0.82] [19]. Compared with placebo, canagliflozin lowered by 20% the risk for non-fatal myocardial infarction (MI), non-fatal stroke or cardiovascular death (HR: 0.80; 95% CI: 0.67–0.95) and by 39% the risk for HF hospitalization [HR: 0.61; 95% CI: 0.47–0.80].

Subsequently, the DAPA-CKD (Dapagliflozin and Prevention of Adverse Outcomes in Chronic Kidney Disease) was a trial that was designed to investigate whether the cardiorenal protection afforded by SGLT-2 inhibitors extends to patients across multiple CKD stages with or without T2DM [20]. In this trial, 4304 patients with eGFR of 25–75 mL/min/1.73 m^2^ and UACR of 200–5000 mg/g were randomized to dapagliflozin (10 mg/day) or placebo. Approximately one-third of study participants had non-diabetic CKD. Once again, DAPA-CKD was stopped early for reasons of efficacy. Over a median follow-up of 2.4 years, dapagliflozin provoked a 39% placebo-subtracted reduction in the primary outcome that was defined as the composite of sustained > 50% decline in eGFR, ESKD, or death from renal and cardiovascular causes (HR: 0.61; 95% CI: 0.51–0.72) [20]. In prespecified subgroup analyses stratified by the diabetic status at baseline, the magnitude of benefit of dapagliflozin on the primary composite outcome was similar in those with (HR: 0.64; 95% CI: 0.52–0.79) or without T2DM (HR: 0.50; 95% CI: 0.35–0.72). The cardioprotective benefit of SGLT-2 inhibition was also confirmed. Compared with placebo, dapagliflozin lowered by 29% the risk for cardiovascular death or HF hospitalization (HR: 0.71; 95% CI: 0.55–0.92). Notably, DAPA-CKD was also the only trial to show a clear benefit of SGLT-2 inhibition on survival since the risk for all-cause death was 31% lower in the dapagliflozin group than in the placebo group (HR: 0.69; 95% CI: 0.53–0.88) [20].

The consistency of the benefit of SGLT-2 inhibitors on cardiovascular and kidney failure outcomes was investigated in an updated 2021 meta-analysis of all 6 currently available phase 3 trials involving a total of 46,969 participants [21]. Compared with placebo, SGLT-2 inhibitor therapy was associated with 10% reduced risk for major adverse cardiovascular events (pooled HR: 0.90; 95% CI: 0.85–0.95), 22% reduced risk for the composite outcome of cardiovascular death or HF hospitalization (pooled HR: 0.78; 95% CI: 0.73–0.84), and 38% reduced risk for kidney failure outcomes (pooled HR: 0.62; 95% CI: 0.56–0.70) [21]. The reduction in the risk for HF hospitalization was the most consistent benefit of SGLT-2 inhibitors across the included trials, whereas significant heterogeneity of associations with outcome was evident for cardiovascular death. The benefit across the drug class was greater in magnitude for an associated improvement in the risk for HF hospitalization and kidney failure outcomes [21].

Although SGLT-2 inhibitors were initially introduced for the management of hyperglycemia, these medications appeared to exert pleiotropic actions that extend above and beyond their glucose-lowering efficacy [22]. In fact, the mechanisms mediating the impressive cardiorenal protection afforded by SGLT-2 inhibitors are not yet fully clear. For example, restoration of tubuloglomerular feedback leading to vasoconstriction of the afferent arteriole and reduction in intraglomerular pressure was repeatedly postulated as the main hemodynamic mediator of long-term stabilization of kidney function in response to SGLT-2 inhibitor therapy [23,24]. The association of the initial eGFR change with the long-term eGFR trajectories was explored in a post-hoc analysis of the CREDENCE trial [25]. An acute eGFR drop of >10% occurred more commonly in the canagliflozin group (45%) than in the placebo group (22%). However, eGFR trajectories over the course of the trial were similar across all initial eGFR change subgroups [25]. Thus, the long-term kidney protective effects provoked by canagliflozin in CREDENCE were irrespective of the initial impact on intraglomerular hemodynamics. Other analyses also showed that the initial eGFR drop in response to SGLT-2 inhibitor therapy is even fully absent in patients with advanced-stage 3b or 4 CKD, but the reduction in albuminuria and long-term stabilization of kidney function persist [26,27]. Therefore, future research is clearly warranted to fully elucidate the hemodynamic and non-hemodynamic mechanisms through which SGLT-2 inhibitors protect both the heart and the kidney.

Despite the fact that treatment with SGLT-2 inhibitors is typically well-tolerated, physicians should be aware of some side effects associated with their use in daily clinical practice [6,12]. Genital fungal infection is by far the most commonly reported side effect of SGLT-2 inhibitors. However, severe infectious complications, such as Fournier gangrene, occur much more rarely [28]. Euglycemic diabetic ketoacidosis is another rare but severe side effect that is mainly seen in volume-depleted patients or in patients with concomitant infection. The US FDA had released a black box warning on canagliflozin on the basis of a nearly 2-fold higher incidence of amputations in the CANVAS program [15]. However, this higher risk for amputations was not confirmed over the course of the CREDENCE trial [19]. In any case, it is important to monitor all patients treated with SGLT-2 inhibitors by performing foot examinations at regular intervals. Similarly, a heightened risk for fractures with canagliflozin was observed in the CANVAS program [15], but not in other large outcome trials with SGLT-2 inhibitors [16,17,19,20]. Another safety concern is that SGLT-2 inhibitors may possibly increase the risk for acute kidney injury by inducing volume depletion, but some recent studies are suggesting the opposite, showing SGLT-2 inhibitor therapy to be associated with a lower incidence of acute kidney injury [29].

Taken together, large trials conducted specifically in CKD patients with or without T2DM provided solid evidence that the addition of an SGLT-2 inhibitor to standard-of-care treatment, including a RAS-blocker, is effective in slowing the progression of kidney injury to ESKD and in improving cardiovascular outcomes. On this basis, the 2020 Kidney Disease: Improving Global Outcomes (KDIGO) guidelines recommend the initiation of SGLT-2 inhibitor therapy for cardiorenal protection in patients with T2DM and CKD, when the eGFR is >30 mL/min/1.73 m^2^ [30]. The strength of this recommendation was labeled as Level 1, and the quality of evidence in support of this guidance was high, graded as Level A [30].

## 3. GLP-1 Receptor Agonists

GLP-1 receptor agonists are incretin drugs that improve glycemic control by enhancing glucose-dependent insulin secretion, delaying gastric emptying, and promoting satiety [31]. Similarly, with SGLT-2 inhibitors, large trials designed to demonstrate their cardiovascular safety showed that four of the FDA-approved GLP-1 receptor agonists were superior to placebo in lowering the risk for major adverse cardiovascular events in high-risk patients with T2DM: liraglutide, albiglutide, semaglutide, and dulaglutide (Table 2) [32,33,34,35,36,37,38,39,40,41]. A 2021 meta-analysis of eight trials comprising 60,080 patients with T2DM provided the most updated data on the effects of GLP-1 receptor agonists on clinical outcomes [42]. Overall, compared with placebo, GLP-1 receptor agonists were associated with a 14% reduction in the risk for non-fatal MI, non-fatal stroke, or cardiovascular death (pooled HR: 0.86; 95% CI: 0.80–0.93), 12% reduction in the risk of all-cause death (pooled HR: 0.88; 95% CI: 0.82–0.94), and 11% reduction in the risk for HF hospitalization (pooled HR: 0.89; 95% CI: 0.82–0.98) [42]. There was no evidence of heterogeneity across GLP-1 receptor agonist structural homology. In addition, treatment-induced improvement in major adverse cardiovascular events was similar in those with or without established atherosclerotic cardiovascular disease (*p*-value for the interaction: 0.18). With respect to the kidney protective effects, compared with placebo, GLP-1 receptor agonists were associated with a significant 21% reduction in the occurrence of the composite outcome of new-onset macroalbuminuria, doubling of serum creatinine, sustained ≥ 40% decline in eGFR, renal replacement therapy, or death from renal causes (pooled HR: 0.79; 95% CI: 0.73–0.83) [42]. However, when a more restrictive outcome of worsening of kidney function (defined as either doubling of serum creatinine or sustained ≥ 40% decline in eGFR) was considered, the benefit of GLP-1 receptor agonists was not statistically significant (pooled HR: 0.86; 95% CI: 0.72–1.02) [42].

Further insights on the kidney protective effects of GLP-1 receptor agonists were provided by a combined analysis of 12,637 patients with T2DM enrolled in the LEADER (Liraglutide Effect and Action in Diabetes: Evaluation of Cardiovascular Outcome Results) and SUSTAIN-6 (Trial to Evaluate Cardiovascular and Other Long-Term Outcomes With Semaglutide in Subjects With Type 2 Diabetes) trials [43]. From baseline to 2 years post-randomization, the placebo-subtracted reduction in albuminuria with GLP-1 receptor agonists was 24% (95% CI: 20–27%). Compared with placebo, semaglutide, and liraglutide significantly slowed the annual rate of eGFR decline by 0.87 and 0.26 mL/min/1.73 m^2^/year, respectively. Notably, treatment-induced improvement in the eGFR slope was greater in the subgroup of patients with eGFR < 60 mL/min/1.73 m^2^ than in those with preserved kidney function at baseline. Compared with placebo, therapy with semaglutide or liraglutide provoked a 14% (HR: 0.86; 95% CI: 0.75–0.99) and 20% (HR: 0.80; 95% CI: 0.66–0.97) reduction in the risk for sustained 40% and 50% declines in eGFR during follow-up, respectively [43]. Similarly, directional but not statistically significant reductions were evident for the risk of sustained 30% and 57% decline in eGFR. Once again, when the analysis was restricted to patients with eGFR of 30–60 mL/min/1.73 m^2^ at baseline, the renoprotective benefit of GLP-1 receptor agonists was greater in magnitude (HR: 0.71, 0.67, 0.56, and 0.54 for sustained 30%, 40%, 50%, and 57% decline in eGFR, respectively) [43]. Taken together, this exploratory analysis of LEADER and SUSTAIN-6 trials suggests that the renoprotection afforded by GLP-1 receptor agonists is likely more pronounced in patients with T2DM and pre-existing CKD than in those with preserved kidney function.

The first trial to explore the safety and efficacy of GLP1-RAs in patients with T2DM and moderate-to-advanced CKD was the AWARD-7 (Dulaglutide versus insulin glargine in patients with type 2 diabetes and moderate-to-severe chronic kidney disease) [44]. In this multi-center, open-label trial, 577 patients were randomly assigned to once-weekly injectable dulaglutide 1.5 mg, once-weekly dulaglutide 0.75 mg or daily insulin glargine as basal glucose-lowering therapy. The mean eGFR of patients enrolled in AWARD-7 was 38.1 mL/min/1.73 m^2^; 39% and 44% of participants had micro- and macroalbuminuria at baseline, respectively. Over 52 weeks of follow-up, eGFR remained stable with dulaglutide 1.5 mg and dulaglutide 0.75 mg but declined with insulin glargine. At the study-end, the albuminuria-lowering effect of dulaglutide 1.5 mg and dulaglutide 0.75 mg did not significantly differ from that of insulin glargine [44]. However, in the subgroup of patients with macroalbuminuria at baseline, GLP1-RA therapy provoked a dose-dependent reduction in UACR. The proportional reduction in UACR over the course of the trial was significantly higher with dulaglutide 1.5 mg as compared with insulin glargine (between-group difference: −29.0%; 95% CI: −43.0% to −11.5%). In contrast, treatment-induced reduction in UACR did not significantly differ between dulaglutide 0.75 mg and insulin glargine (between-group difference: −12.3%; 95% CI: −29.0% to 8.5%) [44]. Despite the fact that AWARD-7 was not originally designed to explore between-group differences on kidney events, the proportions of patients with adjudicated CKD progression were numerically (but not statistically significantly) lower in the dulaglutide 1.5 mg and dulaglutide 0.75 mg groups than in those treated with insulin glargine [44].

The effects of GLP1-RAs on “hard” kidney failure and cardiovascular outcomes, specifically in patients with T2DM and CKD are currently under investigation in the ongoing FLOW (A Research Study to See How Semaglutide Works Compared to Placebo in People With Type 2 Diabetes and Chronic Kidney) trial [45]. This phase 3 trial is planning to randomize 3508 patients to double-blind treatment with a once-weekly injectable semaglutide or matching placebo. According to the eligibility criteria, FLOW trial will enroll T2DM patients with eGFR of ≥50 to ≤75 mL/min/1.73 m^2^ and UACR ≥ 300 to <5000 mg/g or patients with eGFR ≥ 25 to <50 mL/min/1.73 m^2^ and UACR 100–5000 mg/g. These patients will receive optimized background therapy with maximal tolerated doses of a RAS-blocker. The prespecified primary efficacy endpoint was defined as the composite of persistent ≥ 50% decline in eGFR, ESKD, or death from renal and cardiovascular causes. Effects of semaglutide relative to placebo on the occurrence of non-fatal MI, non-fatal stroke, or cardiovascular death will be explored as a prespecified secondary efficacy endpoint [45]. The estimated completion date of FLOW trial is on August 2024.

As in the case of SGLT-2 inhibitors GLP-1 receptor agonists represent an incretin-based class of anti-diabetic medications that also protect the heart and the kidney, a pleiotropic action that can only partially be explained by the treatment-induced improvement in glycemic control [6,12,31]. GLP-1 receptor agonists are effective in improving several cardiometabolic risk factors, inducing a reduction in body weight, small decreases in systolic BP, and improvement in serum lipid profile [6,12,31]. With respect to their renoprotective properties, experimental studies have shown that the GLP-1 receptor is expressed in glomerular, tubular, and vascular cells, generating, therefore, the hypothesis that these drugs may act by directly inhibiting the mechanisms that promote the injury to the diabetic kidney. This notion is further supported by animal studies showing that GLP-1 receptor agonists may alter intraglomerular hemodynamics and down-regulate tissue infiltration by non-resident inflammatory cells, production of pro-inflammatory cytokines, adhesion molecules, and reactive oxidative species [46,47,48,49].

The mechanism of action of GLP-1 receptor agonists is complementary to that of SGLT-2 inhibitors and, therefore, combination therapy may offer an additive benefit on cardiovascular and kidney failure outcomes as compared with each therapy alone. Some evidence to support this therapeutic approach was recently provided by the AMPLITUTE-O trial (Effect of Efpeglenatide on Cardiovascular Outcomes) [34]. In this trial, 4076 patients with T2DM, who had either a history of cardiovascular disease or current CKD (defined as an eGFR of 25 to <60 mL/min/1.73 m^2^) plus at least one additional cardiovascular risk factor, were randomized in a 1:1:1 ratio to receive once-weekly injectable efpeglenatide at doses of 4 or 6 mg or placebo. Notably, randomization was stratified according to the use of an SGLT-2 inhibitor at baseline, a drug class that was administered in >15% of study participants as background anti-diabetic therapy [34]. Over a median follow-up of 1.8 years, compared with placebo, efpeglenatide lowered by 27% the primary composite outcome of non-fatal MI, non-fatal stroke, or death from cardiovascular and undetermined causes (HR: 0.73; 95% CI: 0.58–0.92). Efpeglenatide also provoked a placebo-subtracted reduction of 32% in a secondary kidney outcome, defined as the composite of new-onset macroalbuminuria or sustained worsening of kidney function (HR: 0.68; 95% CI: 0.57–0.79) [34]. In prespecified subgroup analyses, the benefit of efpeglenatide on cardiovascular and kidney outcomes was similar and irrespective of the SGLT-2 inhibitor use [34], providing indirect evidence that GLP-1 receptor agonists and SGLT-2 inhibitors may act in a synergistic manner. This research question is crucial and warrants further investigation in properly-designed randomized trials in the future.

The most frequent side effects associated with the use of GLP-1 receptor agonists are nausea, vomiting, and diarrhea [12,31]. These gastrointestinal side effects are usually occurring over the first 2–4 weeks of treatment and can be ameliorated with the administration of lower initial doses and progressive up-titration of therapy. Hypoglycemia is another common side effect, particularly when GLP-1 receptor agonists are used in combination with insulin treatment [12,31]. Another major safety concern is the increased risk for acute biliary disease, indicating that GLP-1 receptor agonists should be used cautiously in patients with a history of cholelithiasis or pancreatitis [50]. GLP-1 receptor agonists have also been associated with increased risk for medullary thyroid cancer and, therefore, these agents should not be prescribed to patients reporting a relevant medical or family history [12,31].

Collectively, large-scale trials designed to demonstrate the cardiovascular safety of GLP-1 receptor agonists provided strong evidence that this drug class is effective in improving cardiovascular outcomes in patients with T2DM, a benefit that appears to be similar in magnitude with the cardiovascular risk reduction provoked by SGLT-2 inhibitors [51]. Secondary analyses of these trials provided preliminary evidence also supporting a kidney protective effect of GLP-1 receptor agonists that remains to be demonstrated by the ongoing FLOW study, a phase 3 trial designed to test the safety and efficacy of semaglutide specifically in patients with T2DM and CKD. On this basis, the 2020 KDIGO guidelines recommend the use of a long-acting GLP-1 receptor agonist in patients with T2DM and CKD who have not achieved adequate glycemic control despite treatment with metformin and an SGLT-2 inhibitor or when the use of metformin and SGLT-2 inhibitors is contraindicated (i.e., in patients with eGFR < 30 mL/min/1.73 m^2^) [30]. The strength of this recommendation was labeled as Level 1, and the quality of evidence was graded as moderate, Level B [30].

## 4. Conclusions

**Figure 1:**
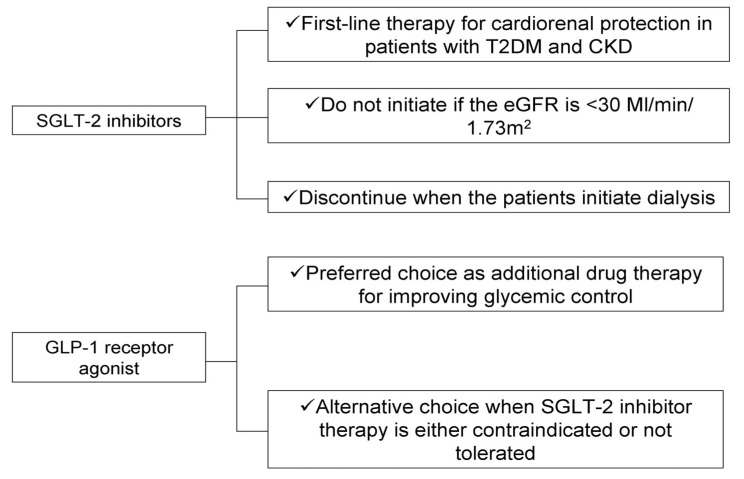
Key clinical practice recommendations for the use of SGLT-2 inhibitors and GLP-1 receptor agonists in patients with diabetic kidney disease.

For almost two decades, the management of patients with T2DM and CKD relied on the optimal glycemic and BP control, whereas RAS-blockade was the only guideline-based treatment proven to be effective in slowing the progression of kidney damage and in improving the risk for adverse cardiovascular events. SGLT-2 inhibitors and GLP-1 receptor agonists are two novel classes of anti-diabetic medications with pleiotropic effects that extend beyond their glucose-lowering efficacy. The beneficial effects of these agents on cardiovascular and kidney failure outcomes have substantially modified the therapeutic algorithm of T2DM in patients CKD (Figure 1), offering hope for more effective mitigation of the high residual cardiorenal risk in this particular patient population.

## Figures and Tables

**Table 1:** Summary of major randomized trials exploring the effect of SGLT-2 inhibitors on cardiovascular and kidney failure outcomes.

Parameter	Clinical Trial					
	CV Outcome Trials				Kidney Failure Outcome Trials	
	EMPA-REG OUTCOME	CANVAS	DECLARE-TIMI 58	VERTIS	CREDENCE	DAPA-CKD
Year	2015	2017	2018	2020	2019	2020
Patient characteristics	T2DM at high CV risk	T2DM at high CV risk	T2DM who had or were at high risk for atherosclerotic CV disease	T2DM and atherosclerotic CV disease	T2DM and albuminuric CKD	CKD with or without T2DM
N	7020	10,142	17,160	8246	4401	4304
SGLT-2 inhibitor	Empagliflozin	Canagliflozin	Dapagliflozin	Ertugliflozin	Canagliflozin	Dapagliflozin
Median follow-up (years)	3.1	2.4	4.2	3.5	2.6	2.4
eGFR (mL/min/1.73 m^2^)	74	76	85	76	56	43
eGFR < 60 mL/min/1.73 m^2^ (%)	26	20	7	22	59	89
UACR < 30 mg/g (%)	60	70	69	58	0	0
UACR 30–300 mg/g (%)	29	22	24	30	0	10
UACR > 300 mg/g (%)	11	8	7	9	100	90
Baseline ACEI or ARB use (%)	81	80	81	81	99.9	98
Primary outcome	CV death, non-fatal MI, or non-fatal stroke	CV death, non-fatal MI, or non-fatal stroke	CV death, non-fatal MI, or non-fatal stroke	CV death, non-fatal MI, or non-fatal stroke	Doubling of serum creatinine, ESKD or death from CV and renal causes	Sustained eGFR decline ≥50%, ESKD or death from CV and renal causes
HR * (95% CI)	0.86 (0.74–0.99)	0.86 (0.75–0.97)	0.93 (0.84–1.03)	0.97 (0.85–1.11)	0.70(0.59–0.82)	0.61 (0.51–0.72)

Abbreviations: ACEI = angiotensin converting enzyme inhibitor; ARB = angiotensin receptor blocker; CI = confidence interval; CKD = chronic kidney disease; CV = cardiovascular; eGFR = estimated-glomerular-filtration-rate; ESKD = end-stage kidney disease; HR = hazard ratio; MI = myocardial infarction; SGLT-2 = sodium-glucose co-transporter type 2; T2DM = diabetes mellitus type 2; UACR = urinary albumin-to-creatinine ratio. * The EMPA-REG OUTCOME and CANVAS were designed as non-inferiority trials but showed superiority of empagliflozin and canagliflozin vs. placebo.

**Table 2:** Summary of major randomized trials exploring the effect of GLP-1 receptor agonists on cardiovascular outcomes.

Parameter	CV Outcome Trials							
	ELIXA	LEADER	SUSTAIN-6	EXSCEL	HARMONY	PIONEER 6	REWIND	AMPLITUDE-0
Year	2015	2016	2016	2017	2018	2019	2019	2021
Patient characteristics	T2DM with a recent acute coronary event	T2DM at high CV risk	T2DM at high CV risk	T2DM with and without established CV disease	T2DM and CV disease	T2DM at high CV risk	T2DM with and without established CV disease	T2DM and either history of CV disease or current CKD
N	6068	9340	3297	14,752	9463	3183	9901	4076
GLP1-RA	Lixisenatide	Liraglutide	Semaglutide	Exenatide	Albiglutide	Semaglutide	Dulaglutide	Efpeglenatide
Median follow-up (years)	2.1	3.8	2.1	3.2	1.6	1.3	5.4	1.8
eGFR (mL/min/1.73 m^2^)	76	80	80	76	79	74	75	72
eGFR < 60 mL/min/1.73 m^2^ (%)	25	25	29	22	23	27	22	32
UACR < 30 mg/g (%)	74	64	NA	79	NA	67	65	54
UACR 30–300 mg/g (%)	19	26	NA	17	NA	33 ^*^	27	46 ^*^
UACR > 300 mg/g (%)	7	10	NA	4	NA	-	8	NA
Baseline ACEI or ARB use (%)	85	83	84	80	82	NA	81	79
Primary outcome	CV death, MI, stroke or hospitalization for unstable angina	CV death, non-fatal MI, non-fatal stroke	CV death, non-fatal MI, non-fatal stroke	CV death, non-fatal MI, non-fatal stroke	CV death, non-fatal MI, non-fatal stroke	CV death, non-fatal MI, non-fatal stroke	CV death, non-fatal MI, non-fatal stroke	CV death, non-fatal MI, non-fatal stroke
HR (95% CI)	1.02 (0.89–1.17)	0.87 (0.78–0.97)	0.74 (0.58–0.95)	0.91 (0.83–1.00)	0.78 (0.68–0.90)	0.79 (0.57–1.11)	0.88 (0.79–0.99)	0.73 (0.58–0.92)

Abbreviations: ACEI = angiotensin converting enzyme inhibitor; ARB = angiotensin receptor blocker; CI = confidence interval; CKD = chronic kidney disease; CV = cardiovascular; eGFR = estimated-glomerular-filtration-rate; GLP1-RA = glucagon-like peptide-1 receptor agonist; HR = hazard ratio; MI = myocardial infarction; NA = not available; T2DM = diabetes mellitus type 2; UACR = urinary albumin-to-creatinine ratio. * Combination of UACR 30–300 mg/g and UACR >300 mg/g.

## Data Availability

Not applicable.
